# CT引导下^125^I粒子植入术对晚期肺癌及肺转移癌的治疗作用

**DOI:** 10.3779/j.issn.1009-3419.2020.103.04

**Published:** 2020-06-20

**Authors:** 一青 王, 林海 朱, 旭 林, 诚 何, 舟 安, 杰 汤, 望 吕, 坚 胡

**Affiliations:** 310003 杭州，浙江大学医学院附属第一医院胸外科 Department of Thoracic Surgery, the First Affiliated Hospital, Zhejiang University School of Medicine, Hangzhou 310003, China

**Keywords:** ^125^I粒子, 晚期肺癌, 肺转移癌, 临床评价, ^125^I seed, Advanced lung cancer, Pulmonary metastatic carcinoma, Clinical assessment

## Abstract

**背景与目的:**

原发性肺癌及肺转移癌均为常见肺部恶性肿瘤，是主要的癌症相关死亡原因。晚期肺癌及肺转移癌以全身治疗为主，局部治疗对全身治疗后肺部残存的顽固性病灶或复发病灶为一种有效治疗手段。^125^I放射性粒子植入作为一种高效的适形放疗，对肺部局限性癌灶具有一定的控制作用。本研究旨在探讨计算机断层扫描（computed tomography, CT）引导下经皮穿刺肺组织间^125^I放射性粒子植入治疗晚期肺癌和肺转移癌的临床效果及安全性。

**方法:**

连续性收集2014年1月1日-2018年11月30日浙江大学医学院附属第一医院胸外科行^125^I放射性粒子植入术治疗的105例晚期肺癌和肺转移癌患者的临床病理资料，术后随访至2019年3月。观察粒子植入术的临床疗效及并发症发生情况。

**结果:**

本研究共纳入105例患者，晚期肺癌患者78例，肺转移癌患者27例。粒子治疗患者术后中位生存时间为395天，术后1年生存率约为78.1%，术后2年生存率约为56.1%。粒子植入治疗晚期肺癌的效果与肺转移癌的效果相当。粒子联合射频消融、微波消融、化疗并没有提高粒子治疗效果。但是粒子联合外放射治疗较单纯粒子治疗具有显著生存劣势。

**结论:**

CT引导下^125^I放射性粒子植入术并发症可控，可以作为晚期肺癌和肺转移癌的一种安全有效的治疗方法。

## Abstract (en)

**Background and objective:**

Primary lung cancer and metastatic lung cancer are common malignant tumors of the lung and are the main cause of cancer-related death. Advanced lung cancer and lung metastatic cancer are mainly treated by systemic therapy, and local treatment is also an effective treatment for the refractory or recurrent lesions in the lungs after systemic treatment. ^125^I radioactive particle implantation, as an efficient conformal radiotherapy, has a certain control effect on localized lung cancer. The purpose of this study was to investigate the clinical efficacy and safety of computed tomography (CT) guided percutaneous puncture of lung tissue with ^125^I radioactive particle implantation in the treatment of advanced lung cancer and lung metastatic cancer.

**Methods:**

Continuouslycollectedthe clinical and pathological data of 105 patients with advanced lung cancer and metastatic lung cancer treated by ^125^I radioactive seed implantation in the Department of Thoracic Surgery, First Affiliated Hospital of Zhejiang University School of Medicine from January 1, 2014 to November 30, 2018. The patients were followed until March, 2019. The clinical efficacy and complications of seedimplantation were analyzed.

**Results:**

A total of 105 patients were included in the study, 78 patients with advanced lung cancer and 27 patients with lung metastases. The median survival time after seed therapy was 395 days. The 1-year survival rate was about 78.1%, and the 2-year survival rate was about 56.1%. Seed implantation for advanced lung cancer is equivalent to lung metastasis. Seed combined with radiofrequency ablation, microwave ablation, and chemotherapy did not improve seed treatment. However, particle combined with external radiation therapy has a significant survival disadvantage compared with simple seed therapy.

**Conclusion:**

The CT-guided ^125^I radioactive seed implantation has controllable complications and can be used as a safe and effective treatment for advanced lung cancer and lung metastases.

肺癌为全球发病率和致死率最高的肿瘤，我国肺癌发病率和死亡率位居各类肿瘤之首^[1]^。在我国近75%肺癌患者初诊时已处于局部晚期或发生远处转移，失去最佳手术机会^[1]^。部分肺癌患者经过标准化治疗后出现复发。肺脏因其解剖特殊性和特殊的肿瘤微环境，为转移瘤发生率最高的脏器，约40%的恶性肿瘤患者在自然病程中发生了肺转移^[2, 3]^。目前晚期肺癌、复发肺癌及肺转移癌的治疗方法主要包括化疗、靶向治疗、免疫治疗等全身治疗，及外放射治疗、射频消融治疗、微波治疗、冷冻治疗等局部治疗，但肺部仍有部分病灶表现顽固或发生复发病灶，寻找控制肺部局部癌灶的有效方法为晚期肿瘤研究热点之一。计算机断层扫描（computed tomography, CT）引导下经皮穿刺肺肿瘤组织间^125^I放射性粒子植入为一种新型近距离放射线治疗肺部肿瘤的方法，其发出的γ射线具有近距离杀死肿瘤细胞的作用，作为一种高效的适形放疗，具有持续杀伤肿瘤细胞且对正常组织损伤较低的特点^[4]^。随着治疗计划系统（treatment planning system, TPS）技术的成熟，应用^125^I放射性粒子治疗肿瘤的临床实践日益增多。然而目前国内对于植入^125^I放射性粒子近距离治疗晚期肺癌和肺转移癌的相关报道较少且缺乏长期疗效数据。本研究对^125^I放射性粒子治疗肺部恶性肿瘤患者的临床疗效和并发症进行回顾性分析，探讨粒子治疗在肺部恶性肿瘤治疗中的有效性和安全性。

## 1 资料和方法

### 1.1 一般资料

连续性选取2014年1月1日-2018年11月30日于浙江大学医学院附属第一医院胸外科接受^125^I放射性粒子植入术治疗的105例晚期肺癌和肺转移癌患者作为研究对象，收集其临床病理资料并门诊复查随访。本研究经医院伦理委员会审批通过并且患者已知情同意。患者纳入标准：经手术或穿刺活检病理确认肺癌或肺转移癌患者；至少经手术切除原发灶治疗和/或一线化疗后肺部仍有癌灶或复发病灶的患者；因疾病原因或身体状态无法行肺切除手术；肿瘤最大直径 < 8 cm；肺部肿瘤数量≤4个；卡氏功能评分（Karnofsky performance status, KPS） > 70分；告知患者及家属^125^I放射性粒子植入治疗注意事项及风险，并签署知情同意书。患者排除标准：大量胸水患者；全身肿瘤广泛转移的患者；有出凝血障碍且难以纠正的患者；有心、肝、脑和肾等重要脏器的严重病变的患者。

### 1.2 治疗方法

纳入患者术前需完成血常规、血凝常规、肝功能和生化等各项检查，强化CT扫描定位肿瘤并将影像数据传送至TPS系统，根据TPS确定^125^I放射性粒子植入的数量及靶区分布。设定处方剂量100 Gy-120 Gy，选用放射活度为0.5 mCi-0.8 mCi的粒子，设计针道，针道间距约为1 cm。患者取合适体位，精神紧张者给予镇静剂，监测生命体征，备好胸腔穿刺包，消毒铺巾，1%利多可因局部麻醉，CT扫描定位选取穿刺点，按照TPS治疗计划布针、植入粒子，手术完成后按压穿刺点20 min。粒子植入后行CT扫描进行剂量验证，剂量不足者立即或择期再次补充植入粒子。术后给予抗感染治疗，术后1 d复查X线胸片检测肺部并发症情况。

### 1.3 术后随访及观察指标

患者术后半年每月医院门诊复查并随访，记录患者复查结果、术后恢复情况、相关并发症和术后治疗情况。之后每3个月门诊复查并随访。对于超过计划复查时间2个月的患者，安排专人进行电话随访。生存期的计算方法为患者首次接受^125^I放射性粒子植入直至末次随访或死亡，本研究随访终止时间为2019年3月。

### 1.4 统计学分析

所有数据采用SPSS 22.0软件（IBM SPSS公司）进行统计分析。分类变量采用例数（*n*）和百分比（%）表示，连续变量采用中位数（全距）表示。生存分析使用*Kaplan-Meier*分析方法及对数秩检验。双尾检验*P* < 0.05表示有统计学差异。

## 2 结果

### 2.1 患者一般特征及并发症情况

本研究共纳入103例接受肺间质粒子植入的患者，其中男性76例，女性27例；中位年龄66岁；晚期肺癌患者78例，肺转移癌患者25例。患者中位住院天数为9 d。肺转移癌组最多的为肝癌10例，其次为结直肠癌6例，食管癌4例，乳腺癌3例，下颌下腺癌和尿道上皮癌各1例。总体患者中双肺多发肿瘤最多，有23例。晚期肺癌组以右肺下叶肿瘤、左肺下叶肿瘤和左肺上叶肿瘤居多，分别为19例、19例和15例。肺转移患者中双肺多发肿瘤患者最多，为14例。最常见并发症为气胸有6例，两例患者行胸腔闭式引流，其他4例保守治疗观察好转后出院。其次为放射性肺炎、胸痛、咳出粒子患者各2例，放射性肺炎及胸痛患者给予对症治疗及抗感染治疗，观察好转后出院。皮下气肿患者1例，未予治疗自行好转。并发症总体发生率为12.6%，在晚期肺癌组为14.1%，在肺转移癌组为8%。术后住院天数中位值晚期肺癌组为8 d，肺转移癌组为12 d。患者一般临床特征及并发症见表 1。

**1:** 晚期肺癌和肺转移癌的临床病理特征 Clinicopathological characteristics of advanced lung cancer and lung metastatic cancer

Characteristic	Total (*n*=103)	Advanced lung cancer (*n*=78)	Lung metastatic cancer (*n*=25)
Gender [*n* (%)]			
Male	76 (73.8)	58 (74.4)	18 (72.0)
Female	27 (26.2)	20 (25.6)	7 (28.0)
Age (yr)	66.0 (28, 87)	66.5 (28, 87)	60.0 (38, 84)
Primary tumor type [*n* (%)]			
Lung cancer	78 (75.7)	78 (100.0)	0 (0.0)
Liver cancer	10 (9.7)	0 (0.0)	10 (40.0)
Colorectal cancer	6 (5.8)	0 (0.0)	6 (24.0)
Esophageal cancer	4 (3.9)	0 (0.0)	4 (16.0)
Breast cancer	3 (2.9)	0 (0.0)	3 (12.0)
Submandibular adenocarcinoma	1 (1.0)	0 (0.0)	1 (4.0)
Urinary tract epithelial cancer	1 (1.0)	0 (0.0)	1 (4.0)
Lung tumor location [*n* (%)]			
Trachea	1 (1.0)	1 (1.3)	0 (0)
Multiple left and right lung lesions	23 (22.3)	9 (11.5)	14 (56.0)
Right lung multiple lesions	7 (6.8)	5 (6.4)	2 (8.0)
Right upper lobe	20 (19.4)	19 (24.4)	1 (4.0)
Right lung middle lobe	2 (1.9)	2 (2.6)	0 (0)
Right lower lobe	10 (9.7)	8 (10.3)	2 (8.0)
Left lung multiple lesions	2 (1.9)	0 (0)	2 (8.0)
Left upper lobe	17 (16.5)	15 (19.2)	2 (8.0)
Left lower lobe	21 (20.4)	19 (24.4)	2 (8.0)
Complication [*n* (%)]			
None	90 (87.4)	67 (85.9)	23 (92.0)
Radiation pneumonia	2 (1.9)	2 (2.6)	0 (0)
Cough out radioactive seeds	2 (1.9)	2 (2.6)	0 (0)
Subcutaneous emphysema	1 (1.0)	1 (1.3)	0 (0)
Pneumothorax	6 (5.8)	4 (5.1)	2 (8.0)
Chest pain	2 (1.9)	2 (2.6)	0 (0)
Length of hospital stay (d)	9.0 (1, 38)	8.0 (1, 34)	12.0 (1, 38)

### 2.2 患者随访及生存

随访期共失访18例患者，失访率为17.5%。截至随访终点，共36人死亡，1例因肺部重症感染死亡，1例因肺气肿死亡，1例因肺栓塞死亡，其他33例均因肿瘤原因死亡。总体患者中位生存时间为13.3个月（0.5个月-66.8个月），患者总体*Kaplan-Meier*生存曲线见图 1。晚期肺癌患者中位生存时间为13.4个月（0.5个月-66.8个月），肺转移癌患者中位生存时间为10.1个月（1.5个月-59.3个月），两组患者总体*Kaplan-Meier*生存曲线见图 2，两组生存无统计学差异（*P*=0.285）。

**1:**
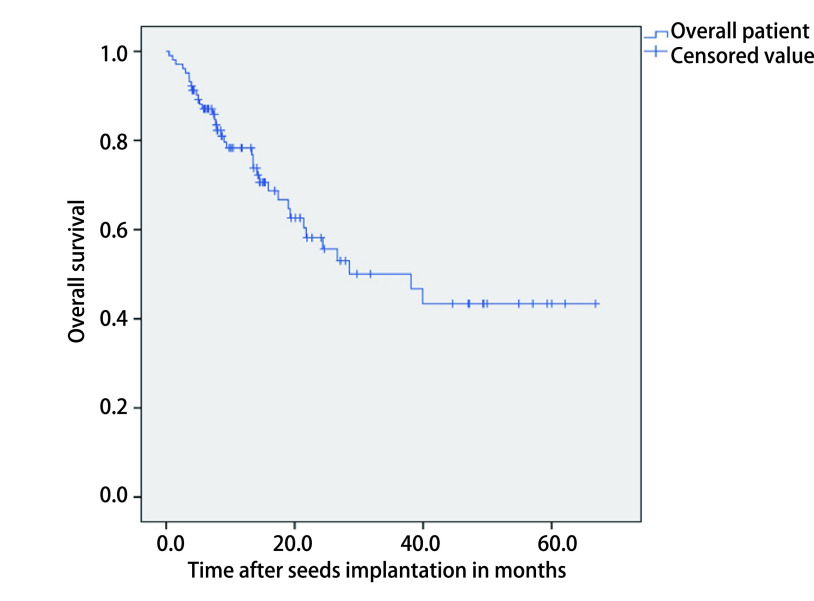
^125^I粒子植入治疗103例患者的总生存期 Overall survival of 103 patients treated with ^125^I seed implantation

**2:**
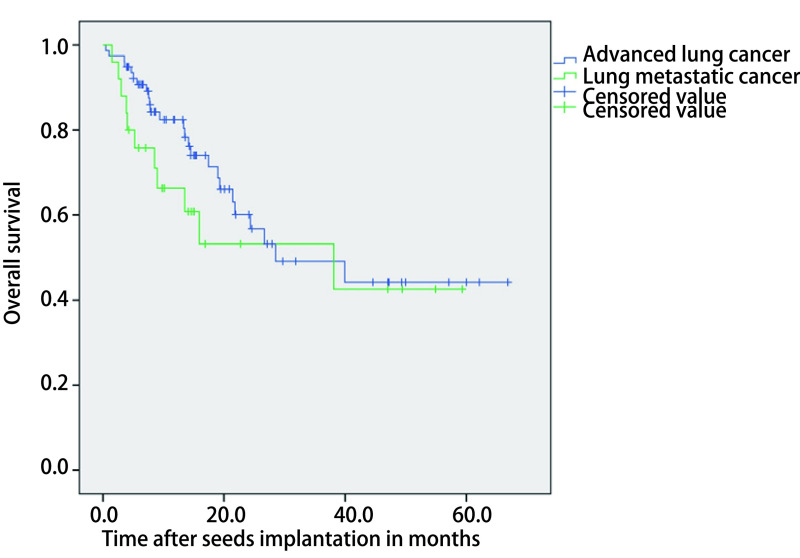
^125^I粒子植入后晚期肺癌和肺转移患者的总生存期（*P*=0.285） Overall survival of patients with advanced lung cancer and lung metastases after ^125^I seed implantation (*P*=0.285)

## 3 讨论

肺癌为我国发病率和致死率最高的恶性肿瘤，随着人口老龄化加重和工业化过程中加重的环境污染，我国肺癌发病率逐年提升^[1]^。由于缺乏早期诊断，近75%患者初次就诊时已处于中晚期，同复发肺癌和肺转移癌一样失去最佳手术机会^[1]^。随着TPS系统的临床应用，^125^I放射性粒子植入术作为一种创伤小且有效的近距离放射治疗方法已广泛用于前列腺癌、脑癌和肝癌等多种肿瘤^[5, 6]^。早在2008年，Martinez-Monge等^[7]^推荐CT引导下^125^I放射性粒子植入术可以作为不能手术的非小细胞肺癌的有效治疗方法。本研究结果显示经粒子治疗患者术后中位生存时间为13.3个月（0.5个月-66.8个月）。粒子治疗对晚期肺癌和肺转移癌起到近似疗效。

气胸为CT引导下^125^I放射性粒子植入术治疗过程中最常见并发症^[8]^。本研究中6例患者发生气胸，2例行胸腔闭式引流治疗好转出院，其他4例未经治疗自行好转。1例患者穿刺点附近皮下气肿，3 d内自行吸收出院。有2例患者出现I级放射性肺炎，经对症治疗后好转出院。有2例穿刺部位疼痛患者，给予止痛治疗后好转。有2例患者出现粒子随痰咳出的情况，咳出的粒子置入铅罐中回收处理。并发症总体发生为12%左右，多为轻度并发症，粒子植入术治疗肺部肿瘤安全可控。

综上所述，CT引导下^125^I放射性粒子植入术可以作为晚期肺癌和肺转移癌的一种有效控制肺部肿瘤的方法，CT引导下肺组织粒子植入治疗并发症总体可控，临床应用安全性较高。
